# The impact of walking on the perception of multichannel electrotactile stimulation in individuals with lower-limb amputation and able-bodied participants

**DOI:** 10.1186/s12984-023-01234-4

**Published:** 2023-08-17

**Authors:** Romain Valette, Jose Gonzalez-Vargas, Strahinja Dosen

**Affiliations:** 1 Department of Health Science and Technology, Aalborg University, Aalborg, Denmark; 2 Ottobock SE & Co. KGaA, Duderstadt, Germany

**Keywords:** Artificial sensory feedback, Lower-limb amputation, Electrotactile stimulation, Prosthesis, Gait

## Abstract

**Background:**

One of the drawbacks of lower-limb prostheses is that they do not provide explicit somatosensory feedback to their users. Electrotactile stimulation is an attractive technology to restore such feedback because it enables compact solutions with multiple stimulation points. This allows stimulating a larger skin area to provide more information concurrently and modulate parameters spatially as well as in amplitude. However, for effective use, electrotactile stimulation needs to be calibrated and it would be convenient to perform this procedure while the subject is seated. However, amplitude and spatial perception can be affected by motion and/or physical coupling between the residual limb and the socket. In the present study, we therefore evaluated and compared the psychometric properties of multichannel electrotactile stimulation applied to the thigh/residual limb during sitting versus walking.

**Methods:**

The comprehensive assessment included the measurement of the sensation and discomfort thresholds (ST & DT), just noticeable difference (JND), number of distinct intervals (NDI), two-point discrimination threshold (2PD), and spatial discrimination performance (SD). The experiment involved 11 able-bodied participants (4 females and 7 males; 29.2 ± 3.8 years), 3 participants with transtibial amputation, and 3 participants with transfemoral amputation.

**Results:**

In able-bodied participants, the results were consistent for all the measured parameters, and they indicated that both amplitude and spatial perception became worse during walking. More specifically, ST and DT increased significantly during walking vs. sitting (2.90 ± 0.82 mA vs. 2.00 ± 0.52 mA; p < 0.001 for ST and 7.74 ± 0.84 mA vs. 7.21 ± 1.30 mA; p < 0.05 for DT) and likewise for the JND (22.47 ± 12.21% vs. 11.82 ± 5.07%; p < 0.01), while the NDI became lower (6.46 ± 3.47 vs. 11.27 ± 5.18 intervals; p < 0.01). Regarding spatial perception, 2PD was higher during walking (69.78 ± 17.66 mm vs. 57.85 ± 14.87 mm; p < 0.001), while the performance of SD was significantly lower (56.70 ± 10.02% vs. 64.55 ± 9.44%; p < 0.01). For participants with lower-limb amputation, the ST, DT, and performance in the SD assessment followed the trends observed in the able-bodied population. The results for 2PD and JND were however different and subject-specific.

**Conclusion:**

The conducted evaluation demonstrates that electrotactile feedback should be calibrated in the conditions in which it will be used (e.g., during walking). The calibration during sitting, while more convenient, might lead to an overly optimistic (or in some cases pessimistic) estimate of sensitivity. In addition, the results underline that calibration is particularly important in people affected by lower-limb loss to capture the substantial variability in the conditions of the residual limb and prosthesis setup. These insights are important for the implementation of artificial sensory feedback in lower-limb prosthetics applications.

**Supplementary Information:**

The online version contains supplementary material available at 10.1186/s12984-023-01234-4.

## Background

A lower-limb amputation is a dramatic event, that has a major impact on the quality of life [1]. Increasing mobility is a regularly expressed need by people with lower-limb amputation [2, 3], and this has motivated a steep improvement in prosthetics technology [4–6]. However, despite the development of microprocessor-controlled passive and powered devices, important differences are still present between prosthesis users and the general population during balancing and walking [4, 7–12]. These differences are at least partially due to the lack of somatosensory feedback: the disruption of control and somatosensory pathways after an amputation has been associated with a lowered capacity to produce rapid gait adjustments after perturbation and the ability to generate forces [7–14]. Indeed, the studies in the literature demonstrated that artificial somatosensory feedback could improve walking and balance, by improving gait symmetry and normalcy, as well as facilitate the feeling of embodiment and reduce phantom limb pain [15, 16].

The feedback can be provided using invasive [17–24] or non-invasive stimulation [25–60] to convey the prosthesis state to the user (e.g., knee angle [17–19, 30, 39–41, 56], foot contacts [17–21, 28, 29, 31–39, 41–44, 49–52, 54, 58–60]). Invasive techniques create vivid phantom limb sensations but require additional surgeries [17–24]. Visual and auditory interfaces have high fidelity but are limited to lab settings [45–60]. Vibrotactile and electrotactile interfaces are therefore commonly used for compact, wearable solutions [25–44]. In electrotactile stimulation, tactile sensations are elicited by delivering low-intensity electrical current that travels locally through the skin, to activate superficial skin afferents without stimulating deeper tissues (e.g., muscles). This approach is especially suitable for implementing multichannel interfaces, as there are no moving mechanical elements while the electrodes are thin and can be printed in the desired shape, size, and configuration, leading to flexible and compact solutions. Multichannel feedback is particularly important as it can be used to convey more feedback variables or a single feedback variable with a higher resolution [61–69].

However, tactile stimulation requires calibrated interfaces to ensure clear perception without discomfort. A calibration procedure measures psychometric parameters to determine sensitivity to amplitude and/or location changes, and the obtained parameters are then used to control spatial and intensity modulation. For instance, the stimulation is normally modulated between sensation (ST) and discomfort threshold (DT). Another important parameter is the just noticeable difference (JND), as it determines the effective resolution of the feedback channel and thereby the number of amplitude intervals that the subject can discriminate (NDI). When using spatial encoding, in which the information is conveyed by changing the location (active pad), the two-point discrimination (2PD) threshold needs to be assessed. The 2PD is defined as the minimal distance required between two stimulation locations so that they are perceived as separate stimuli (instead of a single stimulus), and this parameter, therefore, characterizes the spatial resolution of electrotactile feedback [70–74].

It is well established that the psychometric parameters vary across subjects [64, 75], stimulation sites, skin conditions, electrode types, and waveforms [76], and even across studies. For instance, in 1970’ Solomonow et al. systematically measured the 2PD across a range of body locations and stimulation modalities [70, 71]. Compared to other body parts, the thigh was characterized by one of the largest 2PD (9.88 mm). Other studies reported generally larger values by using different protocols: 24 mm [72], 26 mm [72] and 43 mm [73]. Few studies investigated the spatial psychometric properties of the residual limb of participants with a lower-limb amputation using vibrotactile feedback [77–80]. Spatial discrimination (SD) capacities were evaluated while sitting by modifying the frequency and location of the feedback [80], and it was shown that the socket and liner affect the perception capacities [77–79]. Overall, the participants showed lower location error and faster and more accurate responses when vibromotors were in the inner socket [78].

The systematic assessment of the STs has been performed as well and they showed poorer perception on the proximal leg compared to distal locations [81]. Some studies measured STs in people with lower-limb amputation [7, 82–84], demonstrating that skin sensitivity is also dependent on the etiology of amputation [82]. The subjects that had undergone a transtibial amputation from a dysvascular origin showed decreased ST over their residual limb, while the participants that received amputation following a traumatic event appeared to have a higher chance to develop a hypersensitivity of the residual limb [82].

In addition to the intrinsic variability, the psychometric properties also depend on the type of activity performed during the test. Indeed, the studies of human perception indicated that the perception capacities are modulated when the body parts are involved in a movement [85–90]. More specifically, the movement induces a reduction in the perception (an increase of sensory thresholds), also called “sensory gating”, and this has been registered during both active and passive movement (finger [86, 88], arm [85, 86, 89], and leg[87]). Interestingly, the decrease in sensitivity seems to be dependent on the movement intensity [87] as well as the gait phases [90]. However, these studies were conducted on able-bodied subjects and the impact of movement on the sensitivity in people affected by lower-limb loss has not been investigated before.

Most of the studies that evaluated the impact of feedback in people with lower-limb amputation did not calibrate the stimulation or did not indicate explicitly that such a procedure has been used [27–29, 44]. In more recent studies, the feedback was calibrated while sitting, and the users were allowed to fine-tune the feedback themselves using a portable device when the perception was no longer optimal [39–41]. However, the feedback was initially assessed in a resting position, and the stimulation was not provided on the residual limb inside the socket.

Therefore, while some studies suggest that calibration is necessary during the conditions in which the feedback is evaluated [39–41, 91], this has not been investigated systematically and across different psychometric parameters. In addition, as explained above, the previous studies typically focused on a single parameter (ST/DT [7, 81–84], 2PD [70–74], SD [77–80]) that was measured while the subjects were normally relaxed and sitting, thereby fully focused on the stimulation. However, a single parameter is not enough to characterize perception, which has both intensity and spatial dimension. In the present study, we therefore conducted a comprehensive evaluation of psychometric parameters in able-bodied participants and individuals with lower-limb amputation during walking and compared them to those estimated while sitting. The psychometric parameters were assessed using electrotactile stimuli provided by a 16-channel compact stimulation device. We assessed the parameters characterizing both amplitude and spatial resolution by measuring ST, DT, JND, NDI, 2PD and SD. The hypothesis was that the spatial and amplitude perception capacities will be lower during walking compared to sitting as dual tasking (walking while perceiving stimulation), muscle, and tissue movements would negatively affect the perception. Furthermore, we expected that the socket of participants affected by an amputation would further impact the perception and contribute to the differences between them and the able-bodied participants.

## Methods

### Participants

Eleven able-bodied S1–11 (4 females and 7 males with a mean age of 29.2 ± 3.8 years), 3 participants with transtibial amputation TT01-03 (3 males, 32, 49, and 58 years old), and 3 participants with transfemoral amputation TF01-03 (2 females and 1 male, 56, 70 and 43 years old) were recruited for this experiment. For the able-bodied participants, the inclusion criteria were the following: age from 18 to 70 years old, and the absence of pathologies affecting cognitive capacities and locomotion. The inclusion criteria for the participants with lower-limb amputation were the following: (1) unilateral lower-limb amputation, (2) age from 18 to 70 years old, (3) absence of pathologies affecting cognitive capacities, and (4) the ability to walk at least 5 min without a break. The information about the participants with lower-limb amputation is provided in Table 1. Each participant was introduced to the experiment through an information leaflet and signed an informed consent form before starting the experiments. The experimental protocol was approved by the Research Ethics Committee of Region Nordjylland, Denmark (approval N-20210033).

**Table 1:** Profiles of participants with lower-limb amputation

ID	TF01	TF02	TF03	TT01	TT02	TT03
Weight (kg)	80	63	70	71.3	100	87
Height (cm)	183	170	169	182	188	186
BMI (kg/m^2^)	23.9	21.8	24.5	21.5	28.3	25.1
Sex	M	F	F	M	M	M
Age (years)	43	56	69	32	49	58
Level of autonomy	Independent walker	Independent walker	Comfort cane	Independent walker	Independent walker	Independent walker
Prosthesis use	Daily	Daily	Daily	Daily	Daily	Daily
Amputation type	Transfemoral	Transfemoral	Transfemoral	Transtibial	Transtibial	Transtibial
Prosthesis side	Right	Left	Right	Left	Left	Left
Foot size (EU)	45	40	39	41	44	46.5
Etiology	Traumatic	Congenital	Cancer	Traumatic	Traumatic	Traumatic
Time since amputation (years)	9	56	50	4	7	1
Phantom pain	No	No	Yes	Yes	Yes	No
Phantom sensation	No	No	Yes	Yes	Yes	No
Time since ambulation (years)	8	55	50	4	6	0.5
Residual limb length (cm)	18	19	10	12.5	12	17
Supplementary information	Nothing to declare	Local pain at the end of the residual limb	Osseo-integration since 2011	Retinopathy premature birth (left eye)	Back pain, rod in the femur	Nothing to declare
Knee module	Rheo Knee XC	Rheo Knee XC	C-Leg 4	–	–	–
Ankle–foot module	Proprio Foot	Proprio Foot	Pro-Flex LP Align	Fillaur—All Pro	Pro-Flex LP	Pro-Flex LP
Socket	Northwestern	Northwestern	Osseo-integration	Patellar Tendon Bearing, Supracondylar	Patellar Tendon Kegel;	Patellar Tendon Kegel;
Suspension	Vacuum-assisted socket	Vacuum-assisted socket	Osseo-integration	Vacuum-assisted socket	Vacuum + Pin-lock	Pin-lock

### Experimental setup

**Fig. 1:**
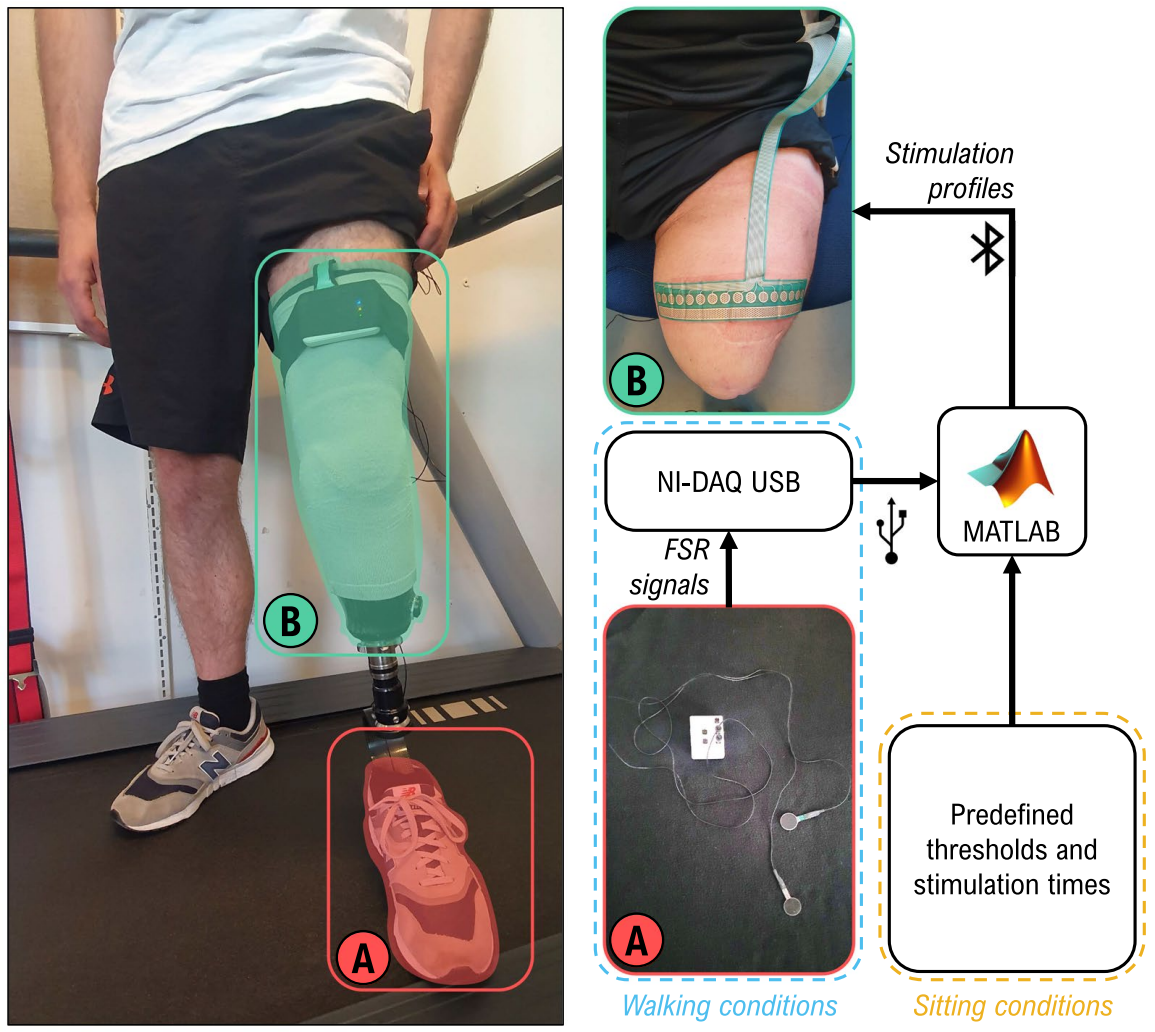
Experimental setup. (**A**) The force sensing resistors (FSRs) placed in the shoe of the ipsilateral side (able-bodied participants) or prosthesis side (participants with lower-limb amputation), and (**B**) the electrotactile device (16-pads electrode) placed on the mid-part of the thigh/residual limb, here displayed on a participant with a transfemoral amputation. The FSRs are used to trigger stimulations at specific moments of the gait cycle during the walking conditions, while the stimulation times during sitting conditions were predefined (see “Amplitude perception assessment” and “Spatial perception assessment” sections)

**Fig. 2:**
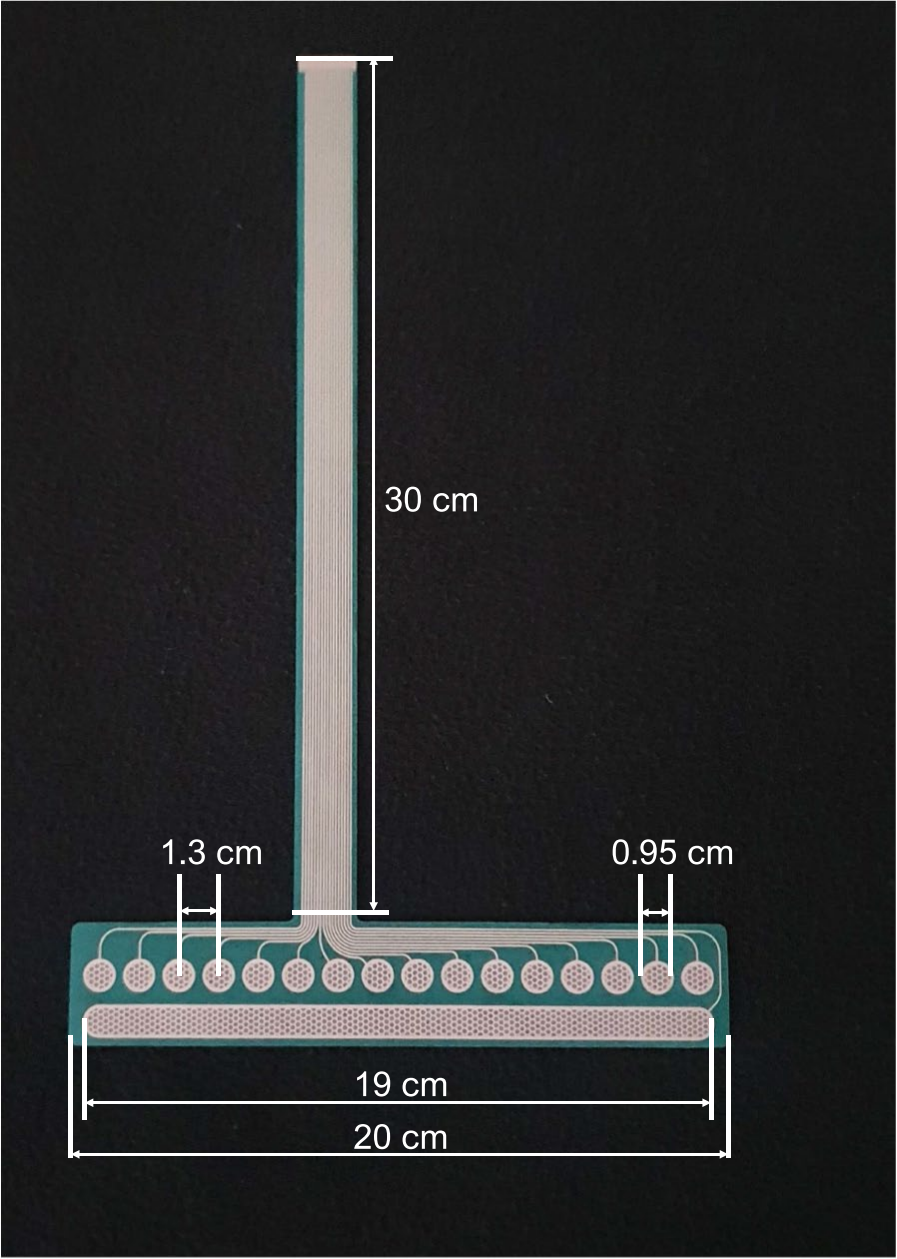
16-pads electrode used in this study. The electrode is composed of 16 circular active cathodes (0.95 cm diameter and 1.3 cm distance between centers, called “pads”), and one common anode (19 cm long). A 30-cm long lead was designed to connect the electrode placed in the socket to the stimulator attached outside of the socket in participants with lower-limb amputation

The experimental setup is shown in Fig. 1. A compact stimulator (MaxSens, Tecnalia, Spain, Fig. 1B) was used to provide electrotactile stimulation [61, 62]. The device is fully programmable and allows delivering stimulation via independently controllable channels using customizable flexible electrode arrays (Fig. 2). The “electrode” contains 16 “pads”, and was made of a polyester layer, an Ag/AgCl conductive layer, and an insulation coating covering the leads. To improve the electrode–skin contact, the pads were covered with conductive hydrogel (AG730, Axelgaard, Denmark). The electrode was identical in the design and configuration of the pads to that used in [61, 62] but the lead was longer so that it could be connected to the stimulator placed outside of the socket.

Biphasic symmetric, current-controlled pulses were generated by the device and the stimulation parameters were controlled by a desktop PC connected to the device via a Bluetooth link. The stimulation parameters, namely, pulse width and amplitude could be modulated online independently for each pad, while the frequency was a global parameter common to all channels. More specifically, the pulse width could be changed from 50 to 1000 µs, through increments of 10 µs; frequency from 1 to 400 Hz, in steps of 1 Hz; and amplitude from 0 to 10 mA, in 0.1 mA increments. In the present study, pulse width, and frequency were constant and set to 500 µs and 50 Hz, respectively, while pulse amplitude was modulated to control the intensity of elicited sensations without ever exceeding 10 mA imposed by the hardware limitations. The pulse width and frequency parameters have been previously used to deliver electrotactile stimulation [61, 62, 92], while the range of amplitudes used in the present study was within that reported in the literature [93, 94].

To time the delivery of stimulation according to the gait phases, a custom-made sensorized insole with 2 force-sensing resistors (FSRs—LuSense, PS3, Standard 174,, Fig. 1A) positioned under the heel and the fifth metatarsal bone was placed in the participants’ shoe on the ipsilateral/prosthesis side. A data acquisition board (NI-DAQ USB-6343, National Instruments) was used to trigger the electrotactile stimuli based on the FSR signals sampled at 1000 Hz. The control of the electrotactile stimuli during sitting and walking was implemented in MATLAB R2021b (Mathworks, USs).

### Experimental protocol

#### Overview

**Fig. 3:**
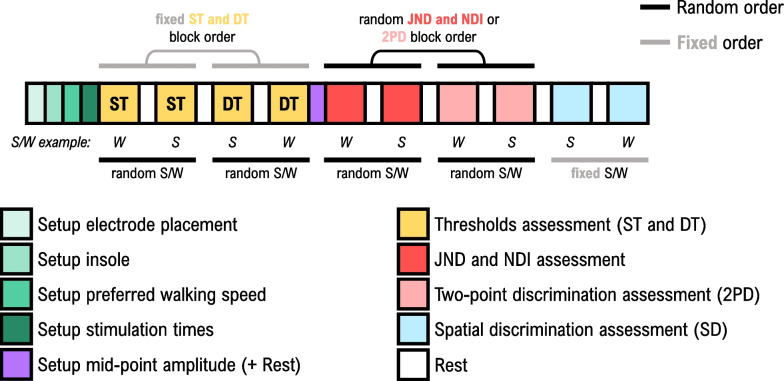
Timeline of the experimental protocol. The term “fixed” denotes fixed order (grey lines) while the term “random” indicates random order (black lines) of the assessments and conditions. After performing the thresholds assessment, the participant was randomly allocated to the JND and NDI or 2PD assessment. For all the assessments, the sitting (“S”) and walking (“W”) conditions were ordered randomly, with the exception of the last block, where the order was fixed (SD assessment). Annotations: "ST, DT": sensation and discomfort threshold; "JND": just noticeable difference; "NDI": number of distinct intervals; "2PD": two-point discrimination threshold; "SD": spatial discrimination; "S": sitting; "W": walking

The present study aimed to assess the psychometric properties of the thigh of able-bodied participants and the residual limb of participants with lower-limb amputation during sitting and walking. The timeline of the experimental protocol is shown in Fig. 3. The session lasted approximately 2.5 h, including the setup of the equipment, tests, and breaks between each condition. Breaks lasted for at least 5 min to ensure that the participants were rested and that no sensation habituation was induced due to the fast succession of conditions [93]. During the sitting condition, the participant was seated on a chair with the knee at approx. 90° of flexion, relaxed and focused on the elicited tactile sensations. Participants with lower-limb amputation wore their prostheses in the same way they wore them during the walking conditions. During the walking tests, the participant walked on a treadmill at the preferred speed determined before starting the assessment. The tests conducted during both sitting and walking included the assessment of amplitude (ST, DT, JND and NDI) and spatial (2PD and SD) perception. The preparation and individual tests are described in the following sections.

The order of the conditions (walking and sitting) and the tests in each condition were randomized across participants as indicated in Fig. 3. The only exception was the SD assessment, which was always performed at the end, as the performance in this task might depend on the amount of previous exposure to the feedback. At the beginning of the experiment, each participant was introduced verbally to the stimulation setup and each of the psychometric tests. Before each test, an example of the task was provided to ensure that the participant understood the verbal description properly.

#### Preparation

First, the skin was cleaned using alcohol swabs and the hydrogel was moisturized to improve the electrode–skin interface. Then, the electrode was placed on the anterior part of the thigh/residual limb (mid-length) by aligning the central pad with the long axis of the limb. This positioning was selected considering that the anterior thigh was identified as a good area to perceive feedback [80] and that the mediolateral orientation was previously used for providing feedback in lower-limb prostheses [31]. For the participants with transfemoral amputation, the electrode was placed as described, except for TF03 which had an osseointegrated system. For the participants with transtibial amputation, the electrode was placed on the thigh, under their liner. In both cases, the flat connector was pulled out of the socket/liner and connected to the stimulator, which was securely attached to the socket/liner. For the able-bodied participants and TF03, the electrode was placed on the thigh while the stimulator was securely attached proximally to the skin. In all the cases, the electrode and stimulator were secured with kinesiology tape. For TF03, the electrode was placed slightly higher on the residual limb with respect to the other participants, as this was the only available location. To optimize the placement, low-intensity pulses were delivered through each pad (1 mA) and increased gradually to a max of 4 mA to check if the participant felt uncomfortable or radiating sensations when activating each pad. In case they reported such sensations, the electrode was moved a few millimeters and the test was repeated until the undesired sensation disappeared. Only subjects S3 and S7 faced this problem, which was solved by slightly moving the electrode. The electrode was then secured using kinesiology tape, and the custom-made foot insole was inserted in the shoe on the prosthesis side in participants with amputation and on the ipsilateral side in able-bodied participants.

The participants were then invited to walk on the treadmill to setup the preferred walking speed and stimulation times. It was indicated to the participants that the preferred walking speed should reflect walking at a comfortable pace with the least effort. To determine the preferred speed, the participants started to walk at a slow treadmill speed (0.14 m/s) and the speed was then increased by 0.028 m/s increments. The display of the treadmill was hidden from view and the participants were therefore unaware of the current walking speed. Once the participant indicated their preferred speed, 0.42 m/s was added to that value, and the speed was decreased to 0.028 m/s to re-establish the preferred speed. This process was repeated 3 times, and the average of the determined speeds (rounded to the nearest decimal) was taken as the preferred speed [94].

**Fig. 4:**
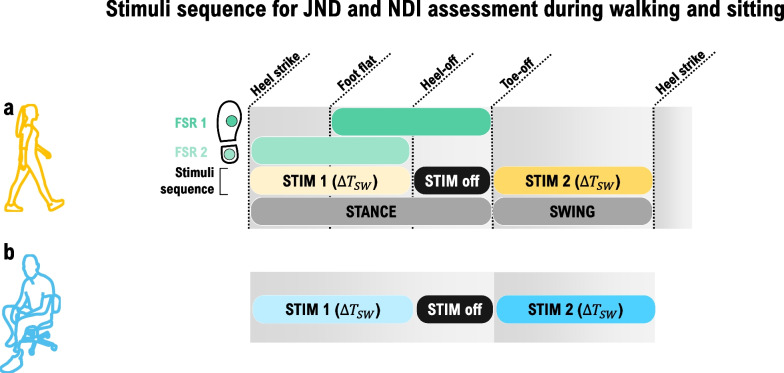
Definition of the stimulation sequences for the amplitude (JND and NDI) assessment, during walking (**a**) and sitting (**b**). The parameter $\Delta {T}_{SW}$ denotes the duration of the swing phase. During the walking condition, the stimulation activation and deactivation were triggered by the gait events, whereas during sitting, the times were predefined and corresponded to those measured beforehand during the initial walking trial (see "Preparation"). STIM 1 and STIM 2 correspond to the first and the second stimuli that are compared by the participant, while STIM off indicates that no stimulation occurs during that time. FSR 1 and 2 are the two force-sensing resistors placed on the foot sole

**Fig. 5:**
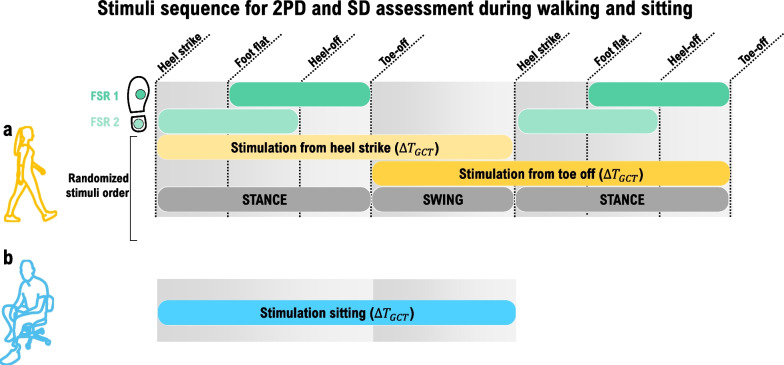
Definition of the stimulation sequences for the 2PD and SD assessments, during walking (**a**) and sitting (**b**). The parameter $\Delta {T}_{GCT}$ denotes the duration of the gait cycle. As in the amplitude perception assessment, the stimulation activation and deactivation were triggered by the gait events during walking, whereas during sitting, the times were predefined and corresponded to those measured beforehand during walking (see "Preparation"). FSR 1 and 2 are the two force-sensing resistors placed on the foot sole

After that, the participants were asked to walk on the treadmill at the preferred speed, and the gains of the two FSRs were manually tuned to ensure that heel strike and toe-off were detected. The participants were then asked to walk on the treadmill at the preferred speed, and 15 gait cycles were recorded to determine the stimulation times to be employed during the experiment in the sitting condition. This has ensured that the times were comparable to those used during the walking condition, in which the stimulation was triggered dynamically by the gait events. The recorded FSR signals were used to determine the heel strike, foot flat, heel-off, and toe-off events as well as the gait cycle duration ($\Delta {T}_{GCT}$), stance ($\Delta {T}_{STA}$) and swing ($\Delta {T}_{SW}$) time. The stimulation timings used during walking and sitting conditions are depicted in Fig. 4 for the JND/NDI assessment, and Fig. 5 for the spatial perception (2PD and SD) assessment, and explained in detail later in the text.

#### Amplitude perception assessment

The amplitude perception assessment aimed to evaluate the ability of the participant to discriminate the changes in the stimulation amplitude during sitting and walking. This information is important for designing encoding schemes that rely on amplitude modulation (e.g., conveying ground reaction forces through the intensity of stimulation).

##### Threshold assessment

After setting up the system, the thresholds were determined. The ST and DT correspond to the minimal stimulation amplitude necessary for the user to perceive the elicited sensation and the intensity at which the sensation feels uncomfortable, respectively. The ST and DT were assessed using the ascending method of limits [95]. This was done once for each pad by increasing the pulse amplitude in steps of 0.1 mA until the participant indicated that he/she felt the stimulation (ST) and that the stimulation became uncomfortable (DT). The ST during sitting/walking was always assessed before DT, to avoid potential habituation due to higher stimulation amplitude. The initial amplitude was set to 0 mA and ST when testing ST and DT, respectively. The average ST and DT across pads were computed for each participant and used as the overall ST and DT for that participant.

The mid-point amplitude (MA) was defined as the midpoint between ST and DT, and this value was selected to generate a clear but not uncomfortable sensation. Once the MA was obtained for each pad, the amplitude was additionally fine-tuned across pads to ensure that every pad elicited similar sensation intensity. The fine-tuning procedure involved activating two adjacent pads sequentially and asking the participant if they perceived the sensations of equal intensity. If this was not the case, the amplitude of the pad with the lower perceived intensity was increased in small steps (0.1 mA) until the participant reported that the intensity became the same (or vice versa for the pad with the higher perceived intensity, depending on what was more comfortable for the participant). This process was then repeated for each pair of adjacent pads.

##### JND and NDI assessment

The JND corresponds to the minimum change in amplitude that is required to perceive a difference in intensity between the two consecutive stimuli. The NDI is computed using JND and represents the number of amplitude levels that can be distinguished by the participant within the full dynamic range (i.e., [ST, DT]) and thereby characterizes the resolution of perception.

The JND and NDI during sitting and walking were assessed over the same stimulation range, which was defined as $\mathrm{MA}\pm 0.5\cdot\mathrm{MA}$**,** where $\mathrm{MA}$﻿ was evaluated during sitting. The percentage of overlap of this stimulation range with the dynamic range (i.e., [ST, DT]) measured during sitting and walking was then determined for each pad, and the pads were then ranked based on the largest overlap. The pads for which the defined stimulation range was bigger than the dynamic range during sitting or walking were discarded, as it indicated that the stimulation would reach both below ST and above DT. The best of the remaining pads according to the ranking was then selected and used as the stimulation channel to determine the JND and NDI.

For the selected pad, we compared 16 equally spaced amplitude levels (8 higher than the reference and 8 lower than the reference) to the reference amplitude ($\mathrm{MA}$) using a method of constants within a two-interval forced-choice task [95]. The amplitudes were selected from the range $\mathrm{MA}\pm 0.5\cdot\mathrm{MA}$, and each comparison was performed 10 times. The stimulation was delivered sequentially and in a pseudorandom order at the two amplitudes, and the participant was asked to report which stimulus (first or second) was perceived as stronger.

The timing and duration of stimulation were defined as explained in Fig. 4. The stimulation duration corresponded to the swing time ($\Delta {T}_{SW}$). During walking, the first stimulation (STIM 1, in Fig. 4a) was triggered by the heel strike, and the second (STIM 2, in Fig. 4a) by the toe-off. During sitting, the first stimulation (STIM 1, in Fig. 4b) was triggered by the experimenter and lasted for $\Delta {T}_{SW}$. The stimulation was then off during $\Delta {T}_{STA}-\Delta {T}_{SW}$ (STIM off, in Fig. 4a, b), and after that, the second stimulus (STIM 2, in Fig. 4b) was delivered for $\Delta {T}_{SW}$. The timing ($\Delta {T}_{SW}$ and $\Delta {T}_{STA}$) was determined in the initial walking trial (as described in the “Preparation”) and the sequence, therefore, mimicked the timing during the walking condition (Fig. 4). The swing time ($\Delta {T}_{SW}$) was selected for the stimulation duration as it is shorter than the stance time ($\Delta {T}_{STA}$). The stimulation was delivered using the same timing during both sitting and walking to prevent potential differences in perception caused by different stimulus duration, directly or due to related phenomena (e.g., habituation).

To determine the JND, a sigmoid function was used to fit the normalized success rates obtained by comparing 16 amplitudes [96]. The point of subjective equality represents the amplitude at which the two consecutive stimulations are perceived as equal (50% of the likelihood range). The JND represents the minimal change in amplitude (in % of the amplitude range) that the participant perceives in 75% of cases. The JND was therefore computed by subtracting the point of subjective equality from the amplitude obtained at 75% of the likelihood range.

Finally, the JND was used to compute the NDI that can be perceived by the participants during sitting and walking. The NDI can be computed using the recursive formula [97]:

1 $${\mathrm{I}}_{\mathrm{k}+1}\leftarrow {\mathrm{I}}_{\mathrm{k}}\cdot \frac{\mathrm{JND}}{100}+{\mathrm{I}}_{\mathrm{k}},$$

where $\mathrm{I}$ is the stimulation amplitude and $\mathrm{k}$ is the level counter. The parameter $\mathrm{k}$ is initially assigned 1, and ${\mathrm{I}}_{1}$ corresponds to the ST. Consecutively, the next amplitude level is computed based on the JND and the previous level, iteratively until the upper stimulation limit (DT) was reached. When the recursion stops, the parameter $\mathrm{k}$ corresponds to the NDI.

#### Spatial perception assessment

This assessment aimed to determine how well the subjects can perceive the changes in the location of the delivered stimulation. This is important when conveying feedback variables using spatial encoding. For instance, each pad can be associated with a range of values, and the momentary value of the feedback signal is then indicated by the currently active channel. For this approach to be successful, the user needs to be able to identify and discriminate between the stimulation channels placed on different locations of the residual limb.

##### 2PD assessment

The 2PD is defined as the minimal distance between 2 pads required for the subject to perceive them individually when they are activated simultaneously. This parameter determines the maximal spatial resolution that can be achieved by the tactile feedback and is thus important information when designing an electrode (e.g., min distance between the pads) and encoding schemes (e.g., how many pads to skip to generate a discriminable change). To determine 2PD, a reference pad (middle of the electrode) was activated simultaneously with a “tested” pad chosen pseudorandomly to be from 1 to 7 pads far from the reference (Fig. 5), using a method of constants within a two-interval forced-choice task [95]. Each pair of pads (14 in total) was presented 10 times to the participants, and they were asked if they feel sensations in one or two locations. The percent of trials in which the simultaneous stimulations were perceived as coming from 2 distinct pads was then computed for each pad combination.

The timing of stimulation is shown in Fig. 5. The pulse amplitude was set to $\mathrm{MA}$, and the stimulation lasted for an entire gait cycle ($\Delta {T}_{GCT}$). During walking, the stimulation was triggered pseudorandomly by heel strike or toe-off and then deactivated once the same event was detected again. The pseudorandom activation was introduced to eliminate the confounding effect that a specific gait phase might have on perception, and therefore, half of the trials were presented starting from heel strike and half from toe-off. During sitting, the stimulation was triggered by the experimenter and lasted for $\Delta {T}_{GCT}$ that was measured beforehand in the initial walking trial (as explained in the “Preparation”). After each stimulation was delivered, the participant reported the answer, which was registered by the experimenter.

To determine the 2PD, the mean number of pads perceived (1 or 2) for each spacing between the pads was calculated. The value 1 was then subtracted from this mean to obtain the normalized values (0–1), expressing the likelihood to perceive 2 individual pads. The closer the score to 1, the more likely that the stimulation was perceived in two points (rather than one point) by the participant. Subsequently, logistic regression was applied to fit a sigmoid curve to the data obtained in the experiment [98]:

2 $$\mathrm{F}\left(\mathrm{x};\mathrm{\alpha },\upbeta \right)=\frac{1}{1+{\mathrm{e}}^{-\upbeta \left(\mathrm{x}-\mathrm{\alpha }\right)}},$$

where $\mathrm{F}$ is the fitted sigmoid function, with the parameter $\mathrm{\alpha }$ referring to the distance at which $\mathrm{F}=0.5$, thus indicating the distance at which the participants were equally likely to report one or two points. The parameter $\upbeta$ represents the slope of $\mathrm{F}$﻿ at this distance, while $\mathrm{x}$﻿ is the distance in mm. This technique has been used previously to assess 2PD [99, 100]. The 2PD thresholds for sitting and walking were defined as the values corresponding to $\mathrm{F}=0.75$, which corresponds to the distance at which the participants reported that they perceived the stimulation in two points in 75% of all cases.

##### SD assessment

The SD assessment was performed to evaluate the ability of the participant to discriminate individual pads. To ensure a challenging yet feasible task, every second pad was included in the SD assessment. This test comprised 3 phases: familiarization, reinforced learning, and evaluation. Familiarization and reinforced learning were performed during sitting, while the evaluation was performed during sitting and walking, in a balanced pseudo-random order. The stimulation during the walking condition was delivered randomly throughout the gait cycle following the same scheme as in the 2PD test. During the sitting condition, the stimulation duration was equal to $\Delta {T}_{GCT}$ and was triggered by the experimenter.

During the familiarization stage, the pads were first activated in sequence (from #1 to #8), and then in a random order to familiarize the participants with sensations elicited by each pad. The experimenter informed the subject about which pad was activated. After this, the reinforced learning phase was performed: the pads were activated randomly while the participants were asked to guess the location (active pad). Afterward, the correct answer was provided by the experimenter. 24 trials were delivered during a block by activating each pad 3 times in random order. The participants performed at least 5 blocks, with a maximum of 10 blocks. The reinforced learning stopped if the participants reached ~ 80% accuracy, corresponding to 5 miss-localizations. If the participant could not reach the target accuracy after 10 blocks, the highest performance was recorded as the baseline after the training. The last phase was the evaluation conducted following the same procedure as during the reinforced learning, but without providing verbal feedback on the correct answer to the participant. In this phase, the participants performed 80 trials (10 presentations × 8 pads).

To analyze the performance during the SD assessment, the confusion matrix was computed for each participant for the best reinforced learning trial, and the evaluations during sitting and walking. Consequently, the success rate, i.e., the ratio of correct guesses vs. incorrect guesses were computed for each pad, and the average score for each participant was defined as the average success rate across the 8 pads.

### Statistical analysis

The statistical analysis was performed only for the able-bodied group due to the low sample size in the case of participants affected by lower-limb amputation. The normality of the data was assessed using the Shapiro–Wilk test and the tests did not show the evidence of non-normality (p > 0.05). Therefore, a paired t-test was applied to compare the psychometric parameters obtained during sitting and walking conditions. The threshold for statistical significance was set at p < 0.05. The data analysis was conducted in Matlab (R2021b, Mathworks, Natick, MA, USA), and the statistical tests were performed using SPSS 28 (IBM, Armonk, NY, USA).

## Results

### Calibration of the self-selected walking speed and stimulation times

The average self-selected walking speed was 1.02 ± 0.12 m/s for the able-bodied participants, while it was 0.73 ± 0.20 m/s for the participants with lower-limb amputation. The average gait cycle time in able-bodied participants was 1.19 s ± 0.09 s, with an average stance time of 0.74 ± 0.08 s and an average swing time of 0.44 ± 0.04 s. For participants with lower-limb amputation, the average gait cycle time was 1.38 ± 0.20 s, with an average stance time of 0.80 ± 0.10 s and an average swing time of 0.58 ± 0.12 s. The stance percentage (stance time relative to the gait cycle time) was 62.5 ± 3.3% in able-bodied and 58.48 ± 3.1% in participants with lower-limb amputation.

### Amplitude perception assessment

**Fig. 6:**
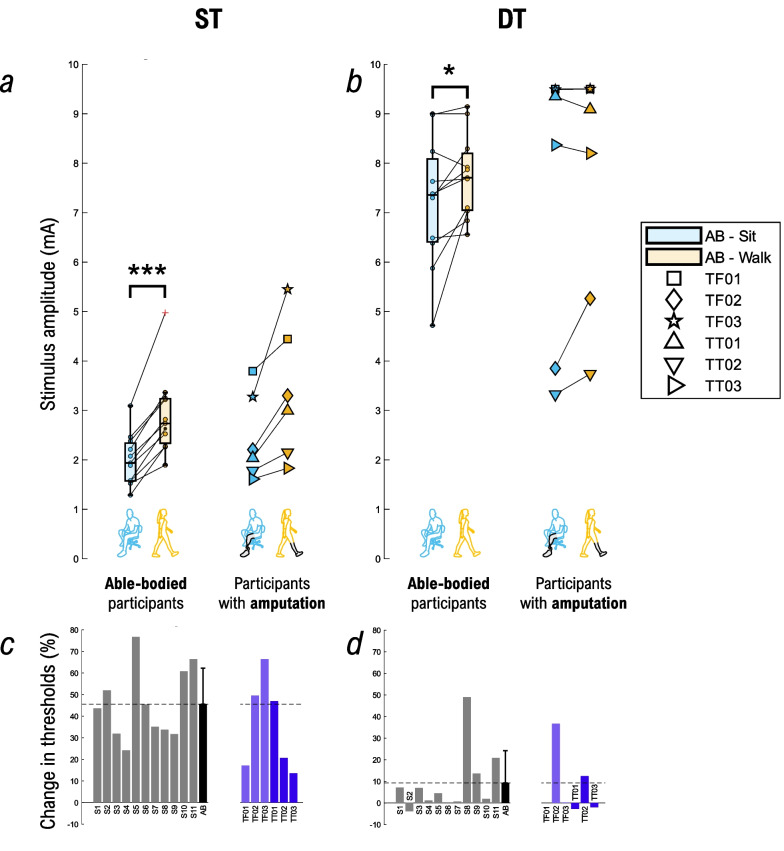
Thresholds assessment (ST and DT). Summary results for the sensation (ST) and discomfort thresholds (DT) while sitting (cyan) and walking (orange). ST (**a**) and DT (**b**) results are displayed using box plots for able-bodied participants (n = 11) while each participant with a lower-limb amputation is shown individually using attributed markers. The percent changes in ST (**c**) and DT (**d**) between walking and sitting are shown for each participant using bar plots. Able-bodied participants are displayed in grey, the black bar is mean ± std for able-bodied participants, while participants with transfemoral and transtibial amputation are shown in purple and dark blue, respectively. A positive value indicates that the thresholds were higher during walking vs. sitting (and vice versa). Annotations: “AB” = Able-body participants; “S#” = Able-bodied participant, followed by the number; “TF#” = Participant with transfemoral amputation, followed by the number; “TT#” = Participant with transtibial amputation, followed by the number; “ST”: Sensation threshold; “DT”: Discomfort threshold; *p < 0.05, ***p < 0.001

Figure 6 shows the results for the ST and DT during sitting and waking. Both ST and DT were significantly higher during walking vs. sitting in the able-bodied group (2.90 ± 0.82 mA vs. 2.00 ± 0.52 mA; p < 0.001 for ST and 7.74 ± 0.84 mA vs. 7.21 ± 1.30 mA; p < 0.05 for DT). For participants with lower-limb amputation, the ST was also consistently higher during walking vs. sitting (Fig. 6c). The changes in the DT were, however, subject-specific. The DT was either higher (TF02, TT02), equal (TF01, TF03), or slightly lower (TT01, TT03) during walking vs. sitting (Fig. 6d). Similar variability in DT is also present in the able-bodied group (Fig. 6c). TF01 and TF03 reached the maximal capacities of the stimulation system in both conditions (9.5 mA), which explains equal values and the absence of change in perception between the conditions.

**Fig. 7:**
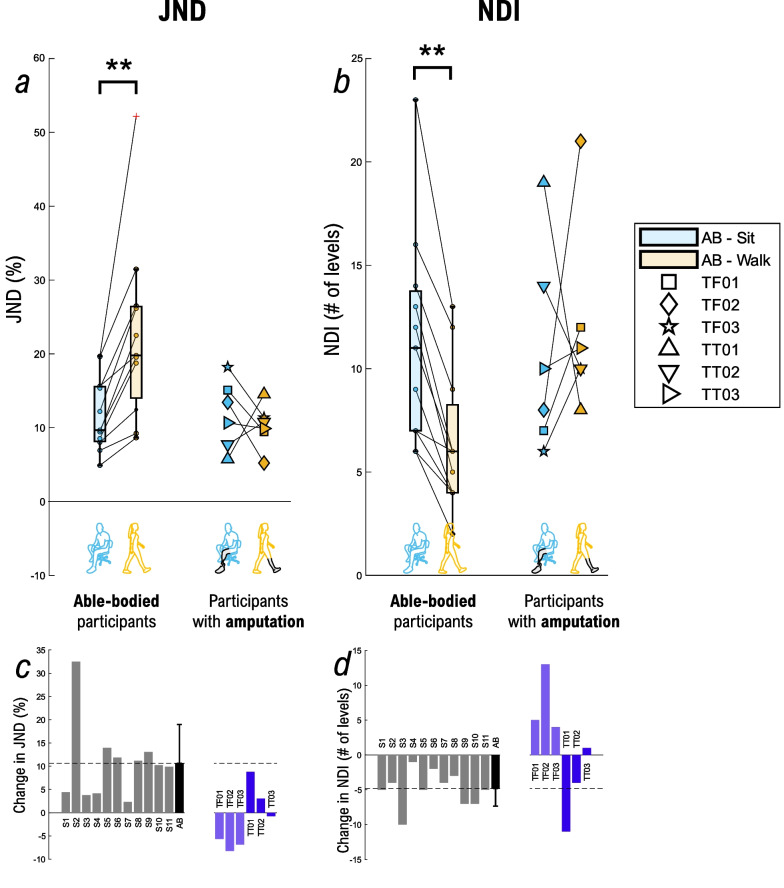
JND and NDI assessment. Summary results for the (**a**) just-noticeable difference (JND) and the (**b**) number of distinct intervals (NDI) while sitting (cyan) and walking (orange). The box plots summarize the results of able-bodied participants (n = 11), while each participant with a lower-limb amputation is shown individually using attributed markers. The change in (**c**) JND and (**d**) NDI between walking and sitting is shown for each participant in the bar plots. Able-bodied participants are displayed in grey, the black bar is the mean ± std for the able-bodied group, while participants with transfemoral and transtibial amputation are in purple and dark blue, respectively. A positive value indicates that the variables were higher during walking vs. sitting (and vice versa). Annotations: “AB” = Able-body participants; “S#” = Able-bodied participant, followed by the number; “TF#” = Participant with a transfemoral amputation, followed by the number; “TT#” = Participant with transtibial amputation, followed by the number; “JND”: just-noticeable difference; “NDI”: number of distinct intervals; *p < 0.05, **p < 0.01

The results for JND and NDI are displayed in Fig. 7. In able-bodied participants, the JND (Fig. 7a) was significantly higher during walking vs. sitting (22.47 ± 12.21% vs. 11.82 ± 5.07%; p < 0.01), which means that during walking, the participants were less sensitive to the change in stimulation amplitude. Interestingly, while the two participants with lower-limb amputation (TT01 and TT02) exhibited the same trend with 8.78% and 3.04% increase in JND during walking, the trend was opposite in the other participants, in whom the JND was lower during walking (decrease in JND of − 5.64% (TF01); − 8.23% (TF02); − 6.85% (TF03); and − 0.77% (TT03), Fig. 7c). In these cases, therefore, walking improved the sensitivity to detect a change in amplitude.

The results for the NDI reflected those for the JND (Fig. 7b). In general, able-bodied participants could discriminate only a few levels between ST and DT and the NDI was lower during walking vs. sitting (average loss of 4.81 intervals; p < 0.01). For the two participants with lower-limb amputation with higher JND during walking, the NDI also decreased (TT01: − 11 intervals; TT02: − 4 intervals), while in the other participants, the NDI increased for 1 (TT03), 4 (TF03), 5 (TF01) and 13 (TF02) levels during walking vs. sitting (Fig. 7d). The average psychometric functions during sitting and walking for the able-bodied population, as well as individual psychometric functions for each participant, are displayed in Additional file 1: Fig. S1, and the results for the goodness-of-fit are displayed in Additional file 1: Table S1.

### Spatial perception assessment

**Fig. 8:**
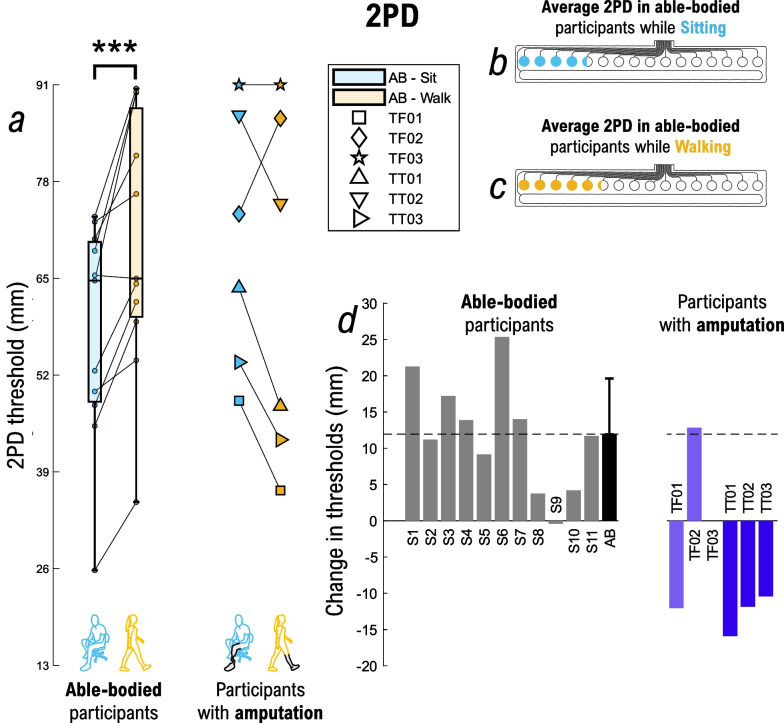
2PD assessment. Summary results for the (**a**) two-point discrimination (2PD) while sitting (cyan) and walking (orange). The box plots summarize the results of able-bodied participants (n = 11), while each participant with a lower-limb amputation is shown individually using attributed markers. Average 2PDs for sitting (**b**) and walking (**c**) in able-bodied participants are displayed on the electrode drawings. The change in 2PD (**d**) between walking and sitting is shown for each participant in the bar plots. Able-bodied participants are displayed in grey, the black bar is the mean ± std for the able-bodied group, while participants with transfemoral and transtibial amputation are in purple and dark blue, respectively. A positive value indicates that the variables were higher during walking vs. sitting (and vice versa). Annotations: “AB” = Able-body participants; “S#” = Able-bodied participant, followed by the number; “TF#” = Participant with a transfemoral amputation, followed by the number; “TT#” = Participant with transtibial amputation, followed by the number; “2PD”: two-point discrimination; ***p < 0.001

The results for 2PD are displayed in Fig. 8. In able-bodied subjects, the 2PD threshold was significantly higher during walking vs. sitting (69.78 mm vs. 57.85 mm; p < 0.001), indicating that a bigger distance between two pads must be considered during walking for the simultaneously active pads to be perceived individually (Fig. 8a). The average difference between the thresholds during walking and sitting was 11.94 mm, which approximately corresponds to 1-pad distance (Fig. 8b, c). Contrary to the results obtained in able-bodied, most of the participants with lower-limb amputation (TF01, TT01, TT02, TT03) perceived the 2 individual points at shorter distances during walking than sitting (Fig. 8d). The exceptions are TF02 whose results are comparable to those of the able-bodied participants, and TF03 who did not reach the 2PD threshold of 75% in either of the conditions. The average psychometric functions for 2PD during sitting and walking for the able-bodied population, as well as individual psychometric functions for each participant are displayed in Additional file 1: Fig. S2, and results for the goodness-of-fit are displayed in Additional file 1: Table S2.

**Fig. 9:**
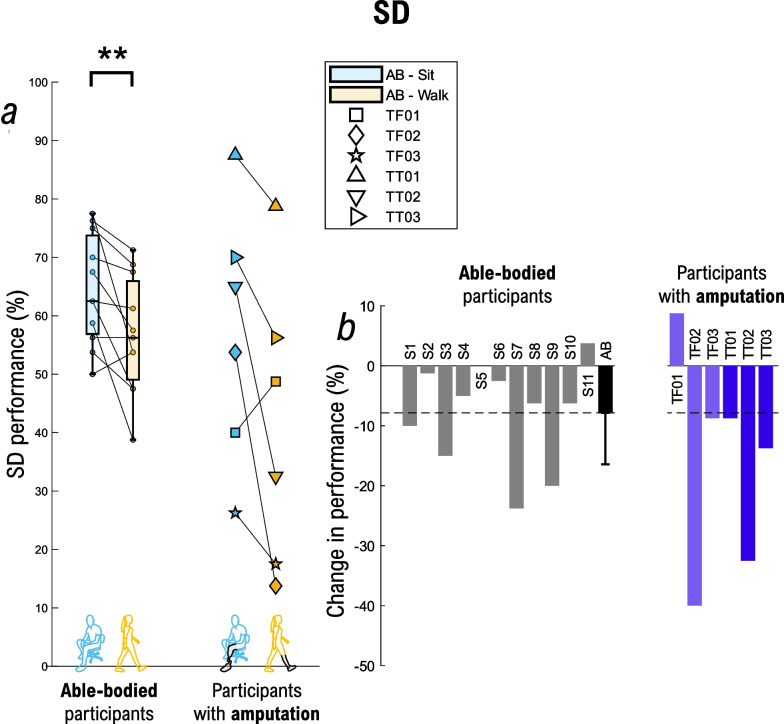
SD assessment. Summary results for the (**a**) spatial discrimination (SD) while sitting (cyan) and walking (orange). The box plots summarize the results of able-bodied participants (n = 11), while each participant with a lower-limb amputation is shown individually using attributed markers. The percent change in SD (**b**) between walking and sitting is shown for each participant in the bar plots. Able-bodied participants are displayed in grey, the black bar is the mean ± std for the able-bodied group, while participants with transfemoral and transtibial amputation are in purple and dark blue, respectively. A positive value indicates that the variables were higher during walking vs. sitting (and vice versa). Annotations: “AB” = Able-body participants; “S#” = Able-bodied participant, followed by the number; “TF#” = Participant with a transfemoral amputation, followed by the number; “TT#” = Participant with transtibial amputation, followed by the number; “SD”: spatial discrimination; **p < 0.01

In the able-bodied group, the performance (Fig. 9a) in SD during walking was significantly lower compared to that achieved in the sitting condition (56.70% vs. 64.55%; p < 0.01). Therefore, it was more difficult for the participants to accurately identify the location of the active pad while they were walking. Five out of the six participants with lower-limb amputation showed similar trends (TF02, TF03, TT01, TT02, TT03) with a decrease in performance of − 40%, − 8.75%, − 8.75%, − 32.5%, − 13.75%, respectively (Fig. 9b). However, TF01 was better during walking with a + 8.75% increase in the success rate compared to sitting. The average confusion matrices for SD during sitting and walking for the able-bodied population, as well as individual confusion matrices are displayed for each participant in Additional file 1: Fig. S3.

## Discussion

Multichannel electrotactile stimulation is an attractive technology to provide feedback to people affected by lower-limb loss because it can lead to compact and low-power solutions, which are convenient for the integration in the socket [61–69]. However, before electrotactile stimulation can be effectively applied, it needs to be characterized using psychometric methods to obtain an insight into the perceptual capabilities of prosthesis users. Although such tests have been conducted in the past, it has not been comprehensively investigated if and how activity level affects perception.

The existing literature proposed optimal procedures to define electrotactile feedback amplitude levels [41], and re-calibration methods to adapt to different general factors known to affect perception (e.g. electrode repositioning, environmental conditions, habituation, etc.) [39, 40]. Some studies mentioned the potential impact of gait on perception in general [91] while some evaluated the impact of gait on amplitude perception but only in able-bodied participants [87, 90]. Therefore, in the present study, we assessed a number of parameters characterizing amplitude (ST, DT, JND, NDI) and spatial perception (2PD, SD), and compared them between sitting and walking in both able-bodied participants and individuals with amputation.

### Influence of gait on amplitude perception

In the able-bodied group, the results are consistent across different measures: the ST, DT, and JND were significantly higher while the NDI was significantly lower during walking. For this group, the results for JND and NDI reflected those obtained for the ST and DT which is a clear indication that walking negatively affects the perception and is in line with results found in the literature. Duysens et al. showed overall higher thresholds in able-bodied participants during walking when transcutaneous nerve stimulation was performed [90], while Paalasmaa et al. indicated that dynamic motion provoked sensory gating [87]. The former study showed an average increase of 134% in ST during walking versus standing, while the latter showed an increase between 26 and 102% during cycling versus sitting [87]. In the present study, the ST was in average approx. 46% higher during walking compared to sitting in able-body participants, and 36% in participants with lower-limb amputation. The NDI in the participants with lower-limb amputation in our study was in the range 6–23 and 2–21 levels during sitting and walking, respectively, while another study indicated between 2 and 5 levels during walking [40]. The difference can be due to multiple reasons (e.g., amplitude vs. pulse width modulation, electrode size and positioning) including the method used. While they applied a quick approach, we used an accurate yet time-expensive method.

The results in the participants affected by a lower-limb loss appear however to be more inconsistent across individuals and measures. Both for participants with transtibial and transfemoral amputation, ST was higher during walking, indicating that sensitivity was negatively impacted by activity, as it was for the able-bodied group. Participants with transtibial amputation appear to follow the trends seen in the able-bodied group (ST, DT, JND increase, and NDI decreases while walking), except for TT03 which shows only minor changes across the conditions. However, for the participants with transfemoral amputation, the results for different measures are not congruent: while they showed higher ST and subject-specific DT, they all exhibited improved perception of the change in the stimulation intensity when walking (JND decreased and NDI increased). A plausible explanation could be the presence or absence of the material placed above and pressing on the electrode. In TT participants, the silicon liner covered the electrode, while TF01 and TF02 wore the socket that applied additional pressure, whereas in TF03, nothing was placed over the electrode (as in able-bodied participants). Importantly, during walking the pressure of the socket changes depending on the gait phase (stance vs. swing) and due to the possible pistoning effect. Additionally, the electrode was placed higher on the thigh of TF03 compared to the other participants, due to the shortness of the residual limb. However, it is less clear why the socket pressure or the position of the electrode would have a different effect on the two parameters (ST and JND).

### Influence of gait on spatial perception

On average, the able-bodied participants demonstrate that spatial perception is reduced during walking, as the 2PD increases and the SD decreases. Participants with lower-limb amputation showed similar trends in the SD assessment as the able-bodied group (SD decreased while walking). It is important to notice that the performance of TF02 and TT02 was especially affected by walking with respect to sitting (− 40% and − 32.5% decrease in success rate, respectively). The SD assessment constitutes a cognitive task, in which the participant must recognize the identity of a pad based on the location of stimulation, but also other cues such as the quality and intensity of sensation (since this will ultimately vary across pads). People with lower-limb amputation show higher cognitive load when walking compared to able-bodied subjects, and this is dependent on the level of amputation, and the type of prosthesis used [101–103]. The results in the SD assessment in participants with lower-limb amputation might therefore reflect the impact of cognitive load, which needs to be considered when designing feedback schemes using spatial encoding (e.g., use fewer pads when producing discrete cues or introduce longer training).

Interestingly, for the 2PD assessment, all participants with transtibial amputation consistently indicated opposite results with regards to the able-bodied group, i.e., while walking, they needed less spacing between the two stimulation points to still perceive them individually. Regarding participants with transfemoral amputation, the results are case-specific: TF01 showed similar results as the transtibial group, while the results in TF02 reflected the trend of the able-bodied group. Finally, TF03 had general difficulties clearly identifying 2 individual stimuli. As in the amplitude assessment, a combination of factors, including the effect of the liner, socket, and electrode placement, might be responsible for the inconsistent results, but this remains to be investigated further. Similarly, as for the amplitude estimation, the results in SD and 2PD in participants with lower-limb amputation appear not to be congruent. Indeed, while participants with transtibial amputation showed better 2PD while walking than sitting, they achieved worse SD performance during walking. In participants with transfemoral amputation, TF01 showed the same trend as the transtibial group, TF02 on the contrary followed a similar trend as the able-body group, while for TF03 the 2PD test did not show the difference between conditions. Importantly, even in the able-bodied group, there are some exceptions in which the 2PD and SD were not consistently related (S5, S9, S11). This discrepancy might therefore reflect the nature of the parameters, which evaluate different aspects of spatial perception. More specifically, while 2PD is an "appearance" based indicator assessing fundamental perception, the SD is a performance-based measure focused on a functional task. This suggests that commonly used methods such as the 2PD should be associated with a performance metric to capture the full ability of the participant to use the feedback device when using spatial encoding. Another potential factor is that the reinforced learning in spatial discrimination provided while sitting might not have been sufficient to properly discriminate the changed sensations during walking. Performing the reinforced learning during walking tasks might increase the performance and make the measures congruent, but this remains to be investigated.

Finally, we can observe substantial differences between participants in 2PD results. Variability is an inherent characteristic of psychometric measurements reflecting individual perceptual capacities and similar variability has been reported in other studies. The methods, test site, gender, test modality, age, and device used, are all factors contributing to variations in the magnitude of the 2PD [70, 71, 104], but also, inherent interindividual and intraindividual variability was observed even when using the same methods and the same site [105].

### Implications for the use of electrotactile stimulation

In summary, the psychometric results obtained in the present study appear to be rather consistent in the able-bodied group where all outcome measures show that the perception is significantly worse during walking compared to sitting. Multiple factors can explain this phenomenon, such as sensory gating due to walking [87, 90], the changes in perception due to the movement of the skin and tissues and/or difficulty in processing sensory information during dual-tasking (perceiving stimulation while walking), where the latter can be especially relevant in prosthesis users who are already challenged with a higher cognitive load [101–103]. Our findings on amplitude perception follow the trends obtained in literature [87, 90], and we further extended those results by indicating that spatial perception is also affected. In participants with lower-limb amputation, however, the results were subject-specific and not always in line with the general trend observed in the able-bodied group. One explanation for the variability of the results in participants with lower-limb amputation and the discrepancy with respect to the able-bodied group is that in the former case many additional factors can affect the perception. Indeed, amplitude perception capacities are modulated by the etiology [82], the increased content in the adipose tissue of the residual limb following amputation due to the disuse of the limb [106, 107] and thickening of the skin and subdermal tissues due to the repeated interaction with the socket [108], but also the modulation in skin temperature inside the socket [109, 110].

Importantly, in the amplitude perception assessment, the changes in the parameters between the two conditions were in some cases quite substantial in both able-bodied participants and participants with amputation. The percent change in ST and DT from sitting to walking could be rather large (up to 76.70% for ST and 49.01% for DT), and likewise, for JND (up to 32.48%) and NDI (up to 13 levels). Similarly, the changes in spatial perception were also quite large for some participants, with up to a 25.36 mm (~ 2 pads distance) increase and a 40% decrease in SD.

These outcomes, therefore, demonstrate that the electrotactile feedback should be calibrated for each participant, ideally in the conditions in which it will be used (e.g., during walking). The calibration during sitting, while more convenient, might lead to an overly optimistic or pessimistic estimate of sensitivity (depending on the participant) when considering the target applications (i.e., walking with a sensate prosthesis). This was confirmed by the verbal reports from the participants. Able-bodied participants indicated during informal discussions that the tests were more difficult to perform during the walking condition and that they had to invest extra effort to properly perceive and interpret the stimulation. In participants with amputation, the verbal feedback was variable and depended on the individual as well as the test performed.

Further research is required to define the proper calibration procedure to compensate for the impaired perception capacities during walking. For instance, one can mount the feedback system, ask the participant to walk on the treadmill or overground and then measure the parameters relevant for the implementation of a specific feedback approach (ST/DT/JND for amplitude modulated and/or 2PD/SD for spatially modulated feedback). After this step, the parameters could be used to calibrate the feedback (determine the number of amplitude and/or spatial levels) and the subjects could then start walking overground and using the prosthesis normally. Such an automatic procedure could be considered both for the clinical setting and out-of-the-lab scenario, in which a participant could define those thresholds quickly using, for example, a mobile phone connected to a prosthesis and stimulation unit.

### Study limitations

Two participants with lower-limb amputation (TF01 and TF03) reached the maximal stimulation current in both conditions (i.e., 9.5 mA for the 16 pads) and still did not report uncomfortable sensations. While this result might seem encouraging at first sight (i.e., the stimulation is well-tolerated), this could also indicate some degree of desensitization of the residual limb. Indeed, for these two participants, the STs were the highest among the participants with amputation and also higher than the average of the able-bodied group in both sitting and walking. Additionally, they exhibited higher JND and lower NDI compared to other participants with lower-limb amputation during sitting, whereas during walking the JND and NDI were comparable to those of other participants.

It is well established that electrotactile stimulation can lead to habituation, but this is most pronounced when the stimulation is constant and prolonged [93]. In the present study, however, the stimuli were relatively short (0.49 ± 0.1 s or 1.26 ± 0.16 s) and they were separated by 2–3 gait cycles of no stimulation and longer breaks of 5–10 min between the conditions to avoid habituation. Therefore, we assume that sensory habituation did not affect the present experiment. This was corroborated by the fact that none of the participants complained that they had difficulties perceiving the stimulation until the end of the experiment.

Another important limiting factor is related to the sample size. On one side, the study demonstrates statistically robust and consistent results across measures in the able-body population, thereby establishing a norm for that population. In contrast, due to the low sample size, the findings obtained in individuals with lower-limb amputation presented as a case-by-case analysis provide only initial insights into psychometric parameter values and associated challenges (e.g., inconsistency across subjects and measures). Nevertheless, the sample size in the present study is within the range normally observed in the literature on artificial sensory feedback for lower-limb prosthetics applications (around 6.44 participants with lower-limb amputation per publication [17–60]). In addition, the present work proposes a systematic approach that can be applied to more participants in the future.

Psychometric measurements are inherently variable, which is a known challenge, particularly when fitting a psychometric curve through a limited set of measurements when the results can be affected also by the chosen fitting method [98]. This variability is clearly visible in our experimental data (see Additional file 1: Figs. S1, S2). Nevertheless, the computed goodness-of-fit indicators (SSE, R^2^, RMSE) imply that the fitted psychometric functions presented sufficiently high performance (low error and high R^2^), especially considering that only some aspects of the curve are important for certain measures (e.g., the estimate of the slope in the case of JND). The best approach to reduce the variability is to increase the number of trials. However, this is constrained by a trade-off between the length of the study, the perception capacities assessed, and the potential influence of other factors that might affect the perception (habituation, fatigue). The focus of the present study was on the systematic assessment of spatial and amplitude perception to establish a general difference between walking and standing. The investigation of other parameters that could further modulate the perception during walking (level of amputation, etiology, age, walking speed, type of activity, type of socket, among others) will be addressed in future work.

## Conclusion

The results of this study indicate that amplitude and spatial perception are subject-specific, especially in persons with amputation, and that they are also significantly affected by activity (walking). Therefore, the overall conclusion is that the electrotactile feedback should be calibrated in the context in which it is likely to be used, as calibrating the feedback device in a resting/sitting condition might not be representative of the perceptual requirements for effective feedback in another condition and vice versa. The further step is to test a larger sample of participants with lower-limb amputation and to increase the range of conditions to fully capture the modulation of psychometric parameters. The results of the present study highlight the importance of context-dependent calibration for the implementation of artificial sensory feedback in lower-limb prosthetics applications.

### Supplementary Information

**Additional file 1.** Individual gait parameters and psychometric results.

## Data Availability

The authors confirm that the data supporting the findings of this study are available within the article and its supplementary materials. Further data that support the findings of this study are available from the corresponding author, SD, upon reasonable request.

## Abbreviations

2PD: Two-point discrimination threshold
AB: Able-body participants
$\Delta {T}_{GCT}$: Gait cycle time
$$\Delta {T}_{STA}$$: Stance time
$$\Delta {T}_{SW}$$: Swing time
DT: Discrimination threshold
FSR: Force sensing resistor
JND: Just noticeable difference
NDI: Number of distinct intervals
SD: Spatial discrimination
ST: Sensation threshold
TF: Participant with transfemoral amputation
TT: Participant with transtibial amputation
