# Supplementary material for: The impact of walking on the perception of multichannel electrotactile stimulation in individuals with lower-limb amputation and able-bodied participants
Source: J Neuroeng Rehabil. 2023 Aug 17;20:108. doi: 10.1186/s12984-023-01234-4 (PMC10436512; doi:10.1186/s12984-023-01234-4)
Supplement: Supplementary file 1 — Additional file 1. Individual gait parameters and psychometric results. [file 12984_2023_1234_MOESM1_ESM.pdf]

Supplementary Materials

Individual gait parameters and psychometric results

Romain Valette[1], Jose Gonzalez-Vargas[2], Strahinja Dosen[1]

[1] Department of Health Science and Technology, Aalborg University, Aalborg, Denmark.

[2] Ottobock SE & Co. KGaA, Duderstadt, Germany

Correspondence: Strahinja Dosen, Department of Health Science and Technology, Aalborg University, Aalborg, Denmark

Email: sdosen@hst.aau.dk

Fig. S1 – JND and NDI results, overview of the individual psychometric functions	Page 2
Table S1 – JND and NDI results, overview of the goodness-of-fit	Page 3
Fig. S2 – 2PD results, overview of the individual psychometric functions	Page 4
Table S2 – 2PD results, overview of the goodness-of-fit	Page 5
Fig. S3 – SD results, overview of the individual confusion matrices	Page 6
Table S3 – Individual walking speed and gait properties	Page 7

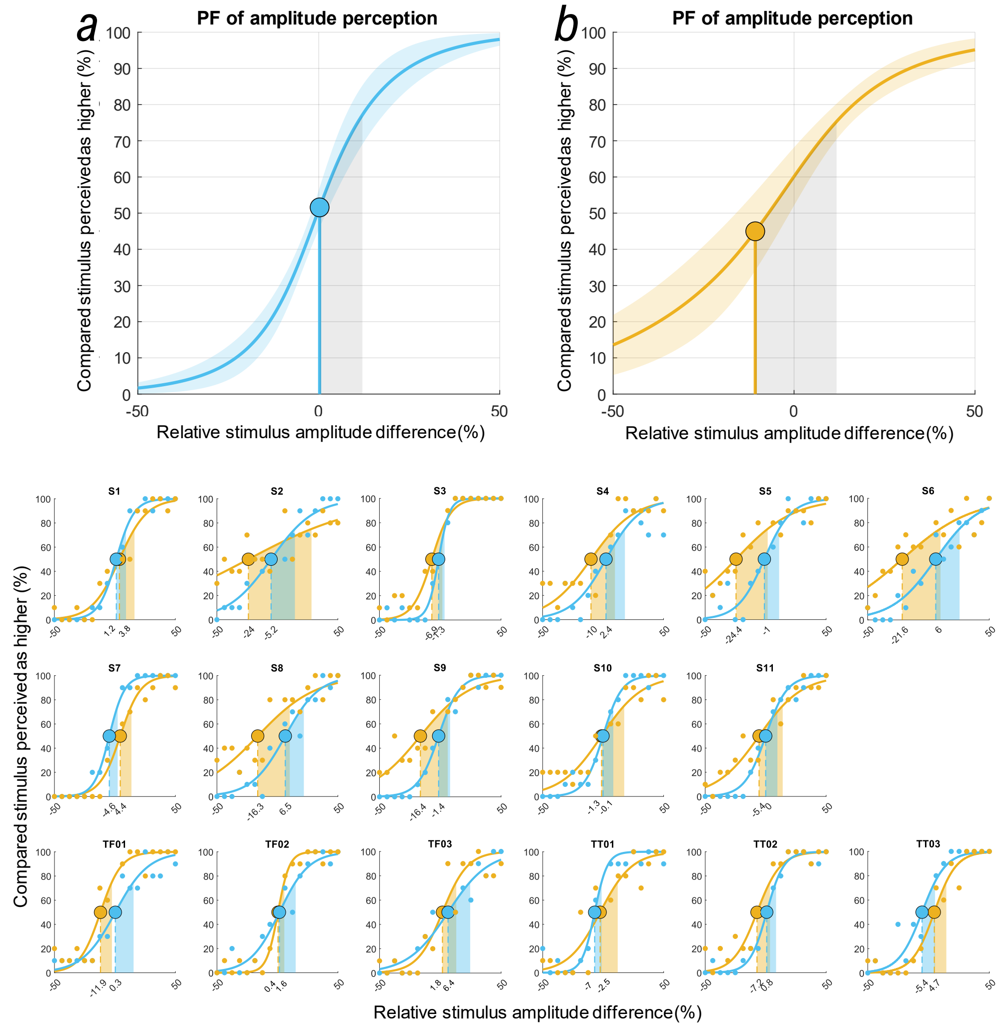

Figure S1 – JND and NDI assessment - Overview of the psychometric functions used to compute the just noticeable difference (JND) and the number of discriminable levels (NDI) during the JND and NDI assessment. The results for sitting are displayed in cyan, and for walking in orange in (a), (b) and (c). The average psychometric functions for the able-bodied population are shown in (a) and (b). Individual results are displayed in (c). “S#” = Able-bodied participant, followed by the number; “TF#” = Participant with transfemoral amputation, followed by the number; “TT#” = Participant with transtibial amputation, followed by the number.

Table S1 – JND and NDI assessment - Overview of the goodness-of-fit results of the psychometric functions obtained during the just noticeable difference (JND) and the number of discriminable levels (NDI) assessment "S#" = Able-bodied participant, followed by the number; "TF#" = Participant with transfemoral amputation, followed by the number; "TT#" = Participant with transtibial amputation, followed by the number; "SSE" = Sum of squared errors; "R^2" = Coefficient of determination; "RMSE" = Root mean squared error.

ID	Sitting			Walking		
	SSE	R^2	RMSE	SSE	R^2	RMSE
S1	0.024	0.992	0.041	0.120	0.954	0.093
S2	0.121	0.936	0.093	0.219	0.621	0.125
S3	0.032	0.991	0.048	0.104	0.962	0.086
S4	0.247	0.880	0.133	0.296	0.828	0.145
S5	0.182	0.925	0.114	0.121	0.880	0.093
S6	0.143	0.922	0.101	0.239	0.760	0.131
S7	0.019	0.994	0.037	0.058	0.980	0.064
S8	0.156	0.931	0.106	0.254	0.801	0.135
S9	0.041	0.986	0.054	0.135	0.902	0.098
S10	0.056	0.981	0.063	0.162	0.905	0.107
S11	0.048	0.983	0.059	0.152	0.917	0.104
TF01	0.133	0.935	0.097	0.195	0.919	0.118
TF02	0.119	0.952	0.092	0.049	0.984	0.059
TF03	0.124	0.939	0.094	0.281	0.888	0.142
TT01	0.093	0.967	0.082	0.371	0.851	0.163
TT02	0.047	0.984	0.058	0.142	0.941	0.101
TT03	0.151	0.947	0.104	0.113	0.960	0.090

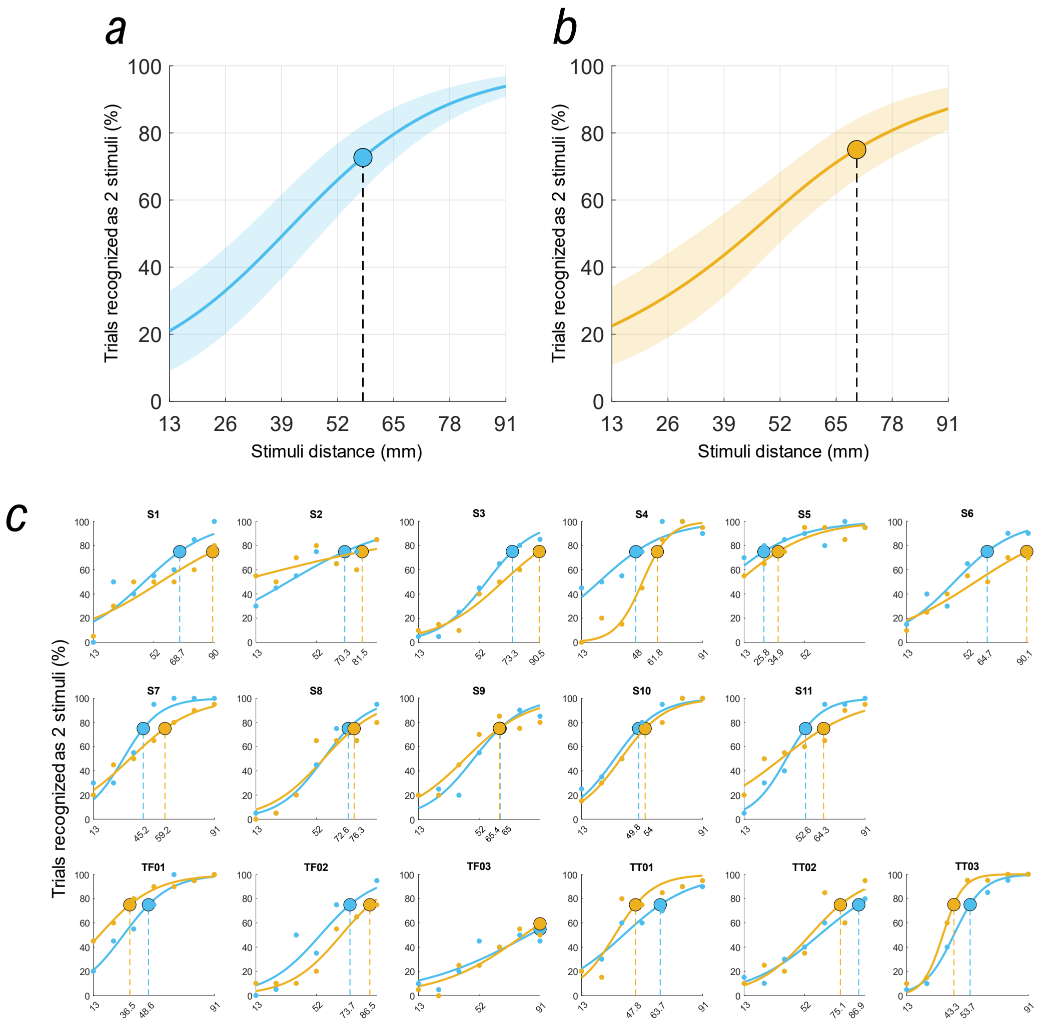

Figure S2 – Two-point discrimination (2PD) assessment - Overview of the psychometric functions used to compute the two-point discrimination (2PD) threshold during the 2PD assessment. The results for sitting are displayed in cyan, and for walking in orange in (a), (b) and (c). The average psychometric functions for the able-bodied population are shown in (a) and (b). Individual results are displayed in (c). "S#" = Able-bodied participant, followed by the number; "TF#" = Participant with transfemoral amputation, followed by the number; "TT#" = Participant with transtibial amputation, followed by the number.

Table S2 - Two-point discrimination (2PD) assessment - Overview of the goodness-of-fit results of the psychometric functions obtained during the two-point discrimination (2PD) assessment. Overview of the goodness-of-fit results for the two-point discrimination (2PD) psychometric functions. “S#” = Able-bodied participant, followed by the number; “TF#” = Participant with transfemoral amputation, followed by the number; “TT#” = Participant with transtibial amputation, followed by the number; “SSE” = Sum of squared errors; “R^2” = Coefficient of determination; “RMSE” = Root mean squared error.

ID	Sitting			Walking		
	SSE	R^2	RMSE	SSE	R^2	RMSE
S1	0.104	0.833	0.144	0.054	0.838	0.104
S2	0.023	0.899	0.067	0.059	0.402	0.109
S3	0.009	0.987	0.042	0.020	0.952	0.063
S4	0.047	0.861	0.097	0.032	0.970	0.080
S5	0.029	0.539	0.076	0.021	0.867	0.065
S6	0.037	0.930	0.086	0.027	0.911	0.073
S7	0.047	0.930	0.097	0.010	0.978	0.044
S8	0.045	0.941	0.095	0.088	0.867	0.133
S9	0.058	0.908	0.108	0.052	0.889	0.102
S10	0.017	0.973	0.058	0.009	0.986	0.043
S11	0.016	0.982	0.056	0.038	0.901	0.087
TF01	0.019	0.968	0.061	0.004	0.985	0.027
TF02	0.112	0.852	0.149	0.025	0.951	0.070
TF03	0.052	0.749	0.102	0.036	0.866	0.085
TT01	0.022	0.950	0.067	0.095	0.859	0.138
TT02	0.018	0.956	0.059	0.111	0.833	0.149
TT03	0.007	0.993	0.037	0.008	0.992	0.040

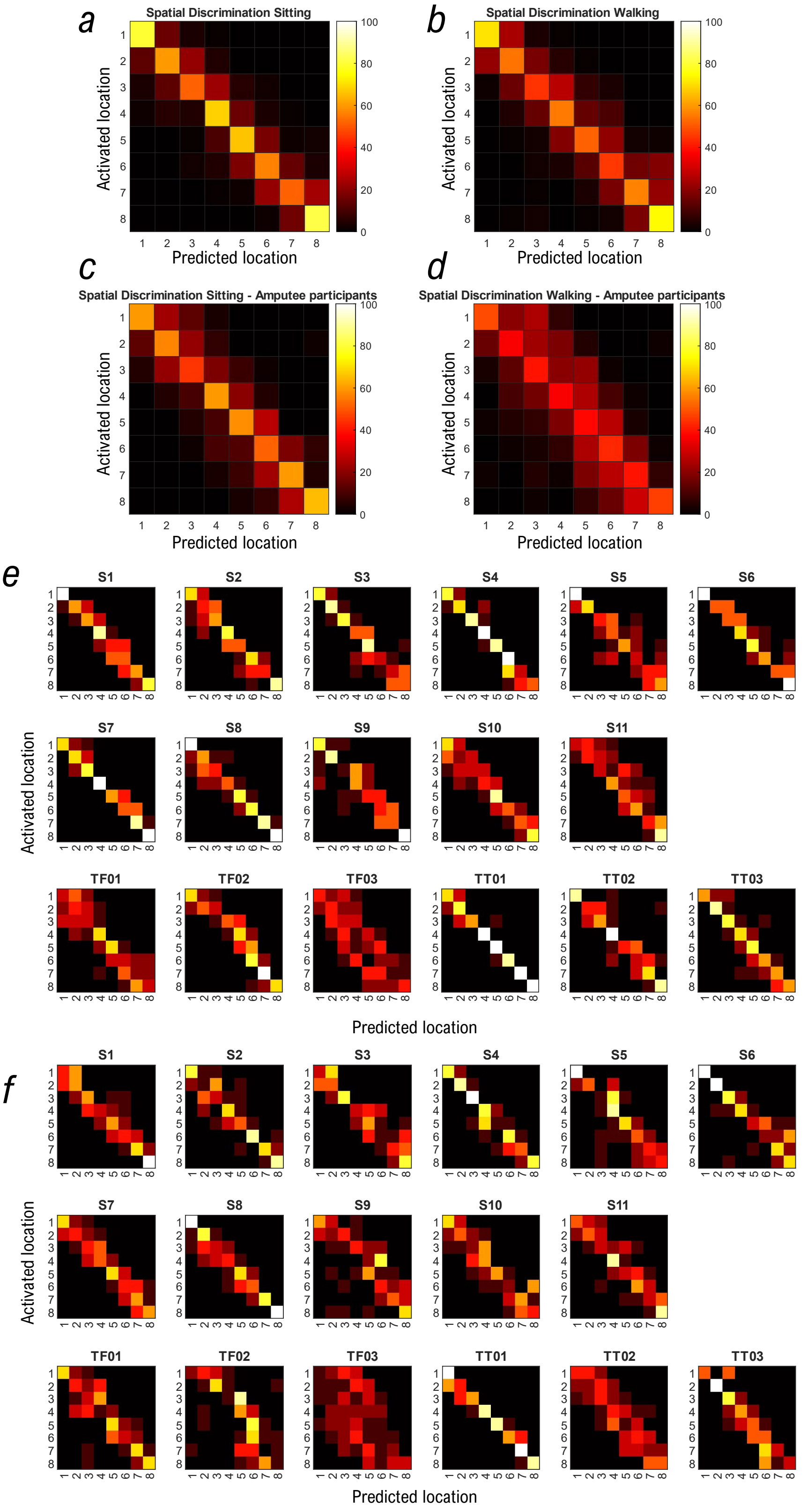

Figure S3 – Spatial discrimination assessment - Overview of the confusion matrices obtained for the Spatial Discrimination (SD) assessment. The average confusion matrix for the able-bodied population is shown in (a) for sitting and (b) for walking. Same for principles for participants with lower-limb amputation in (c) and (d). Individual results are displayed in (e) for sitting and (f) for walking. “S#” = Able-bodied participant, followed by the number; “TF#” = Participant with transfemoral amputation, followed by the number; “TT#” = Participant with transtibial amputation, followed by the number.

Table S3 - Walking speed and gait properties of each participant, used to define the stimulation time for the amplitude and spatial evaluation during Sitting and Walking. Annotations: “AB” = Able-body participants; “AMP” = participants with lower-limb amputation; “S#” = Able-bodied participant, followed by the number; “TF#” = Participant with transfemoral amputation, followed by the number; “TT#” = Participant with transtibial amputation, followed by the number; “ΔT_{STA}” = Stance time; “ΔT_{SW}” = Swing time ; “ΔT_{GCT}” = Gait cycle time.

ID	Walking Speed (m/s)	ΔT_{STA} Stance time (s)	ΔT_{SW} Swing time (s)	ΔT_{GCT} Gait cycle time (s)	Stance percentage (%)	Swing percentage (%)
S1	1.11	0.671	0.399	1.070	62.76	37.24
S2	0.89	0.789	0.517	1.306	56.89	43.11
S3	0.94	0.716	0.473	1.189	60.22	39.78
S4	1.06	0.820	0.436	1.256	65.42	34.58
S5	0.97	0.690	0.404	1.094	63.01	36.99
S6	1.22	0.723	0.445	1.168	61.84	38.16
S7	0.86	0.876	0.448	1.324	65.99	34.01
S8	0.89	0.795	0.420	1.215	65.31	34.69
S9	1.11	0.587	0.488	1.075	54.71	45.30
S10	1.17	0.733	0.414	1.147	63.99	36.00
S11	1.00	0.772	0.438	1.210	63.83	36.17
AB	1.02(±0.12)	0.743 (±0.079)	0.444 (±0.037)	1.187 (±0.087)	62.18 (±3.61)	37.82 (±3.61)
TF01	0.83	0.928	0.764	1.692	55.38	44.62
TF02	0.64	0.734	0.542	1.276	57.39	42.61
TF03	0.42	0.809	0.453	1.262	64.21	35.79
TT01	0.94	0.673	0.484	1.157	58.11	41.89
TT02	0.64	0.892	0.662	1.555	57.29	42.71
TT03	0.89	0.789	0.552	1.341	58.65	41.35
AMP	0.73(±0.20)	0.804 (±0.095)	0.576 (±0.117)	1.380 (±0.202)	58.51 (±3.01)	41.49 (±3.01)
Overall	0.92(±0.21)	0.765 (±0.088)	0.491 (±0.097)	1.255 (±0.163)	60.88 (±3.77)	39.12 (±3.77)
